# Breast Cancer: Exploring the Facts and Holistic Needs during and beyond Treatment

**DOI:** 10.3390/healthcare5020026

**Published:** 2017-05-24

**Authors:** Zhi Xuan Ng, Mei Shan Ong, Tamilarasi Jegadeesan, Shuo Deng, Celestial T. Yap

**Affiliations:** 1 Department of Physiology, Yong Loo Lin School of Medicine, National University of Singapore, Singapore 117597, Singapore; e0005099@u.nus.edu (Z.X.N.); e0013225@u.nus.edu (M.S.O.); phstj@nus.edu.sg (T.J.); 2 National University Cancer Institute, Singapore 1192288, Singapore

**Keywords:** breast cancer, holistic needs, cancer survivorship

## Abstract

Breast cancer patients face challenges throughout the journey of diagnosis, treatment, post-treatment, and recovery. The breast cancer patient is exposed to a multidisciplinary team including doctors, nurses, therapists, counselors, and psychologists. While the team assembled together aims to address multiple facets in breast cancer care, the sub-specialized nature of individual professional practices may constrain the overview of patients’ holistic needs and a comprehensive approach to cancer management. This paper aims to provide an overview of the holistic needs of breast cancer patients at each stage of their cancer journey, addressing their complex physical, psychological, and social needs. As every patient is different, cancer care has to be tailored to each patient based on a holistic needs assessment. This paper also explores how support can be provided from the perspectives of the healthcare providers, family members and caretakers. Examples of general practices at healthcare institutions worldwide as well as supportive care provided by support groups are discussed. The needs of breast cancer patients extend beyond the resolution of cancer as a disease, and the restoration of health as far as possible is a critical component of healing. Understanding the complex issues involved in the journey of breast cancer will aid healthcare providers to be better equipped to sensitively address their concerns and focus on healing the patient holistically. Methodology: This paper provides a literature review of validated practices in different countries and elaborates on the holistic needs of patients at various stages of recovery. This review is based on more than a decade of publications sourced from multiple resources including PubMed journal articles; books and official websites of breast cancer organizations.

## 1. Epidemiology and Classification of Breast Cancer

Breast cancer, a heterogeneous disease in its development and progression, remains the most prevalent female cancer diagnosed worldwide. Based on GLOBOCAN statistics, the incidence of breast cancer has increased from 1.4 million in 2008 to 1.7 million in 2012, which represents a 21.4% increase in the incidence of breast cancer worldwide over this period. Moreover, the incidence of breast cancer was observed to vary across different regions worldwide, whereby breast cancer is more prevalent in developed countries compared to less developed countries, at age-standardized rates per 100,000 of 74.1 and 31.3, respectively. Breast cancer is the leading cause of female cancer deaths worldwide, accounting for up to 15% of all cancer deaths [1,2,3]. Furthermore, mortality has increased steadily from nearly 805 per 100,000 total deaths in 2008 to about 932 per 100,000 total deaths in 2012 [4,5,6,7]. In Singapore, more than 25% of all cancers diagnosed in women are breast cancers, making it the most common cancer among females [4].

Numerous risk factors have been shown to associate with the development of breast cancer and include age, reproductive factors, exo- and endogenous hormonal exposures, personal and family history of cancer, and lifestyle and environmental factors such as alcohol consumption and diet [8,9]. Genetic and epigenetic alterations such as BRCA1/2 mutations have also been shown to affect the risk of lifetime breast cancer [10,11] whereby the risks increase to 65–81% for those with BRCA1 mutation carriers and 45–85% for BRCA2 mutation carriers [12,13]. The classification of breast cancer can be based on both histopathologic and molecular characteristics. Histologically, breast cancer is classified into in situ carcinoma, consisting of ductal carcinoma in situ (DCIS) and lobular carcinoma in situ (LCIS); or invasive carcinoma, which is divided into 7 subtypes [10]. On the other hand, the molecular classification of breast cancer is based on protein expression patterns involving several markers such as estrogen receptor (ER), progesterone receptor (PR), human epidermal growth factor receptor 2 (HER2), HER1 and basal cytokeratin [14]. The differential protein expression gives rise to the molecular classification of breast cancer into different subtypes including luminal A & B, HER2-enriched, basal-like, claudin-low, and normal breast-like [15].

## 2. Diagnosis and Treatment

Screening via diagnostic imaging technologies such as mammogram, ultrasound and magnetic resonance imaging (MRI), and tissue biopsy allows for early detection and diagnosis of breast cancer. Management and treatment of breast cancer, depending on clinical staging, tumor biology, and molecular subtype, include surgery, local tissue-targeting radiotherapy, and systemic therapies such as chemo, hormonal, and targeted therapy. In early stages of cancer, the surgical management of breast cancer via lumpectomy (breast-conserving surgery) or mastectomy (surgical removal of breast tissues) with the removal of clear margins of both invasive and non-invasive cancer, is required. Systemic therapies may be required as post-surgical adjuvant therapy to enhance disease-free and overall survival, depending on the molecular subtyping and pathology determined by tumor and axillary nodal status. In the more advanced stages, systemic therapies are involved in disease control, palliative management, and improvement of overall survival. In terms of disease control, neo-adjuvant systemic therapy, which is the administration of therapeutic agents before a main treatment, is used in the reduction of tumor size and burden, hence impacting subsequent treatment, surgical options, and long-term outcomes [16,17,18]. Throughout the process of detection, diagnosis, management, and treatment of breast cancer, patients experience trauma and stress in physical, psychological, and emotional aspects. Hence, it is important to understand the holistic needs of breast cancer patients to better provide supportive care throughout their journey.

## 3. Holistic Needs

**Figure 1:**
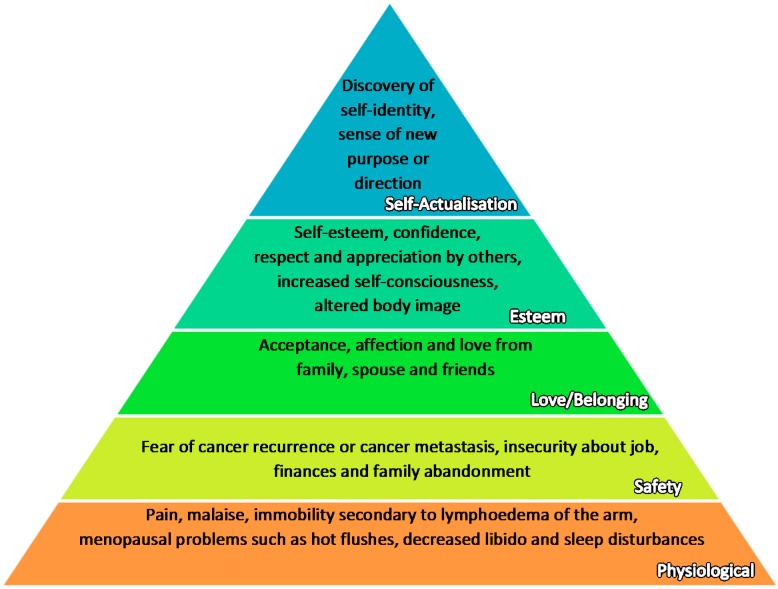
Maslow Hierarchy of Needs applied in the context of breast cancer, with reference from Institute of Medicine (US) (2008), Schmid-Büchi, S. et al. (2008), NCCS Breast Cancer Survivorship Programme (2016).

The well-being of a cancer patient is determined by how well her physical, social, psychological, emotional, and spiritual needs are being met. These needs are also mirrored in the Maslow’s hierarchy of needs, a motivational theory in psychology [19]. The figure below (Figure 1) is modified based on Maslow’s original framework [19] to suit the context of breast cancer patients, based on concerns raised by Institute of Medicine(US) (2008), Schmid-Büchi, S. et al. (2008), NCCS Breast Cancer Surivorship Programme (2016) [20,21,22]. 

It is posited that the more basic needs in the lower tiers must be met before the higher needs can come into focus. However, in the context of cancer therapy, all aspects are assessed and managed concurrently as cancer patients often lose many components of their identity together. Moreover, the distinction between different tiers is interlinked and not as disparate as it may seem. For instance, a strong family support may alleviate debilitating physical pain felt by the patients. 

The concept of cancer survivorship is a widely accepted and well-established notion defined by The National Coalition for Cancer Survivorship, an organization set up by and for cancer survivors in the United States. It defines someone as a cancer survivor from the time of diagnosis to her demise, regardless of whether the cancer is cured, active, or untreatable [23]. It seeks to change the patient’s perspective from that of a passive victim to an active survivor. However, it is worth pointing out that, while this concept serves to create a sense of belonging among cancer patients, some patients may feel uncomfortable being labeled as survivors, especially as it forces them to focus on their disease as a centric theme in life. Such sentiments are echoed by authors including Diana Fields in her autobiography [24].

As each patient at different stages of breast cancer faces varying difficulties and is surrounded by her own unique circumstances, there is a need to individualize a holistic recovery package for every patient based on periodic holistic needs’ assessment. This is a set of recognized assessment tools that allows patients and their healthcare professionals to reflect on their needs and explore how best these could be achieved. It is hoped that patients will be able to better self-manage living beyond breast cancer [25]. 

The National Cancer Action Team in the United Kingdom suggests a number of key stages where a holistic assessment of needs should be conducted [26]. These stages include, at the point of diagnosis; at the commencement of treatment; or at any time when the patient asks for it. These stages correspond to significant milestones along the patient’s journey where needs might change and support is needed.

Before and during the treatment, the cancer care team should explain to the patients and their families about their chances of recovery, treatment benefits and disadvantages, the availability of psychological services, social support and palliative care, and the estimated total costs [27]. Stressing the importance of effective communication in holistic cancer care, the Australian National Breast Cancer Centre has published guidelines on information provision and counseling for breast cancer patients and developed a national communication skills training strategy to train health professionals [28].

Over the past decade, many countries have come to recognize the importance of a multidisciplinary team (MDT) in cancer care. In fact, under the National Health Service in England, it is mandatory for the cancer care team to comprise designated breast surgeons, breast care nurses (BCNs), pathologists, radiologists, and oncologists [29]. Such a multidisciplinary approach has also been adopted by the Swedish Medical Center [30] and the Japan Cancer Institute hospital’s breast oncology center [31]. The efficacy of the MDT is shown by the reduction in mortality in a region of Scotland after the introduction of MDT [29].

Serving as a core member of MDT, a dedicated breast specialist nurse speaks to her patient before and after surgery. For instance, in Singapore, breast care nurses are specially trained to attend to and care for breast cancer survivors, in addition to the surgical oncologist, providing both pre-operative counseling and post-operative rehabilitation [32]. 

The Institute of Medicine (US) [33] also recommends routine post-cancer treatment care for all survivors, in areas such as expected short-term and long-term effects of therapy, post-treatment monitoring for toxicity and recurrence, psychosocial and employment needs as well as preventive lifestyle modifications. A systematic review of studies by the Institute of Medicine (US) has shown that psychosocial interventions in breast cancer can improve quality of life and reduce symptoms of mental illness [34]. Adapting these recommendations into Singapore’s context, the National Cancer Centre Singapore (NCCS) [35] offered a multidisciplinary program that addressed patients’ post-treatment worries, including how complementarily medicines can be incorporated and whether local dishes such as chicken and rice can be consumed. Those who went through the support sessions reported significant improvements in physical symptoms [36]. 

## 4. Holistic Needs at Time of Diagnosis 

For many people, the diagnosis of a malignant breast cancer induces a feeling of dread and fear. Elisabeth Kubler-Ross [37], a Swiss-American psychiatrist, proposed five stages of grief that terminally-ill patients experience. Regardless of whether the diagnosis is terminal or not, cancer patients may experience grief due to a sense of uncertainty and fear of the loss of future [38,39,40]. Patients may also sense a loss of control over their lives as their normal routines are severely disrupted. For instance, a stringent treatment regime and the associated therapy side effects could render them unfit for work. Patients need not necessarily go through the stages chronologically, but can fluctuate through the stages or revert back to any of the initial few stages during the treatment [40]. We constructed Table 1 below based on Elizabeth K. Ross’ original framework on the five stages of grief, with references from Cancer Research UK, to elaborate on the unique challenges faced by breast cancer patients [41].

Apart from facing difficulties in accepting the diagnosis, female breast cancer patients also grapple with self-esteem issues. After the surgery, many of these patients reported feeling less like women [42,43]. They may be concerned that the loss of a breast due to mastectomy or the loss of hair post-chemotherapy would diminish their femininity [44]. Therefore, prior to treatment, care providers play important roles to orientate the patient through the option of breast reconstruction and choose a method of breast reconstruction that suits them best. Care providers should also explore with the patient on how they can help redefine her identity, perhaps by introducing her to other breast cancer survivors and exposing her to a new perspective.

At this stage, cancer patients may feel most vulnerable and insecure as they are at a loss of what to do. In Asian countries, where family plays an integral role in patients’ healthcare, the presence or absence of family support strongly influences patients’ outlook of their disease and their will to fight on. Studies have found that there is a bidirectional effect between a cancer patient’s psychological well-being and her partner’s mental state [45]. Therefore, the healthcare team should ascertain the level of family support, enlist family members’ involvement in the care management, and create opportunities for the family to have meaningful conversations. When delivering the diagnosis, the doctor could sensitively break the diagnosis in phases depending on their patients’ emotional states, and take extra care not to create false hopes. The doctor could also explore goals of care, long-term planning, advance directives, and palliative care with his or her patients if indicated [46]. 

## 5. Holistic Needs during Treatment

### 5.1. Managing Physical Side Effects

Following tumor resection or mastectomy, many patients undergo chemotherapy to eliminate residual cancer cells. Physical side effects of chemotherapy include anorexia, nausea and vomiting, oral ulcers, alopecia, risk of infection, and thrombophlebitis, particularly in patients requiring prolonged intravenous administration of drugs. On the other hand, radiotherapy commonly causes redness and dryness of skin [47]. There are also other long-term complications related to radiotherapy. For example, axillary irradiation may predispose the patient to lymphedema of the arm. If the chest wall is irradiated in left-sided breast cancer, there is also a risk of cardiomyopathy. Other side effects specific to breast cancer patients include osteoporosis, swelling, and immobility of arm and shoulder, thyroid dysfunction, early menopause-associated symptoms (e.g., hot flashes, sleep disturbance, mood changes, vaginal dryness), concerns about fertility, and decreased libido [47]. Some suggestions on how these side effects could be alleviated are provided below.

#### 5.1.1. Professional Advice from Cancer Care Team

With a multitude of information available online and differing advice from well-intentioned family and friends, patients are often at a loss regarding which suggestions to follow. Expert advice provided by the cancer care team on pain relief methods, skin care products, and recommended diets would serve to benefit them. This is validated by recent results from Singapore’s Breast Cancer Survivorship Programme trial [36], where patients reported large improvements in physical symptoms. The American Society of Clinical Oncology Breast Cancer Survivorship Care Guideline has also included the management of physical, psychological and social long-term side effects as one of the five critical areas to address in breast cancer survivorship [48].

#### 5.1.2. Exercises

Targeted exercise is an important part of physiotherapy and rehabilitation for breast cancer survivors. After surgery, patients need to engage in specific exercises to prevent shoulder and arm stiffness. If the surgery involves lymph node removal, the occupational therapist may prescribe exercises to reduce lymphedema as well as manual lymphatic drainage massage, skin care, and compression bandage [49]. On the other hand, non-targeted exercises bring about better immunity, enhanced body awareness, and psychological and physical well-being in breast cancer patients. Exercises including yoga, weight lifting, and jogging have been shown to be effective in reducing anxiety, depression, fatigue, and stress in cancer patients [50,51].

#### 5.1.3. Complementary Medicine

Many people use complementary and alternative medicine (CAM) while they are on conventional treatment. In Singapore, due to its multicultural population, the wide array of CAM encompasses traditional Chinese, Malay, and Indian medicine, health supplements, acupuncture, yoga, and Ayurvedic massage. A cross-sectional study revealed that the use of CAM is highly prevalent among cancer patients in Singapore [52]. Such practice has also been reported in China with the use of Traditional Chinese Medicine (TCM) [53]. An integrative approach between Western treatment and CAM could benefit patients in providing increased overall well-being during breast cancer treatment, by improving the side effects from conventional treatment and enhancing treatment outcomes. However, in order for these benefits to be delivered, it is important for trust to be established between the Western doctor and the patient, so that the patient is forthcoming in telling her doctor what treatment she is receiving. This aids the Western doctor to ascertain whether the CAM would interfere with the conventional treatment, so as to prevent treatment delay and potentially dangerous drug–drug interactions between CAM and chemotherapeutic or radiotherapeutic agents [52]. 

### 5.2. Managing Psychological Stresses

In addition to suffering from debilitating physical side effects, having to take a long break from work brings up another issue of job and financial insecurity, as patients worry about the possibility of losing their jobs. This is complicated by the hefty cost of treatment that can go up to US$23,078 per annum [54]. The added financial and emotional burden of caring for an ill family member may eventually lead to frustration, causing family relationships to deteriorate. Psychologically, this also means that patients can suffer from a fear of abandonment or of being a burden. A sense of self-efficacy could be affected, as they are now dependent on others for not just financial support, but also physical help in getting around. This is compounded by losses such as hair loss during chemotherapy, mastectomy, and other physical changes during treatment, which have led many women to question their self-identity [55]. Therefore, other than linking up patients with relevant social services, an astute breast cancer nurse should also pick up signs of high stress state in patients, such as a change in behavior, as these could indicate a decline into clinical depression. A nurse counseling service can significantly help to reduce distress associated with breast cancer diagnosis and treatment [56]. 

#### 5.2.1. Social Support

An effective social support for cancer patients has been shown by numerous studies to be effective in reducing the negative impact of diagnosis and treatment and promoting psychological well-being [57,58]. The patient’s social support network includes family members, friends, neighbors, other breast cancer patients, and healthcare professionals.

On the contrary, particularly in Asia, where family plays a greater role in healthcare decisions, some patients may find it stressful and confusing when over-anxious family members interfere with every aspect of their daily life, including their diet and habits. To avoid such situations, the American Society of Clinical Oncology recommends that caregivers have an open communication with the cancer patient and give assurance that he or she would be a central part of all discussions and decisions [59]. They should also strive to involve the patient and maintain a sense of normalcy by helping her stay engaged with the world beyond cancer.

#### 5.2.2. Spiritual Care

Patients may find solace and assurance from the knowledge that a higher power exists. The motivational teachings involved in many different religions help to keep their hopes high, diverting attention away from themselves to the religious rituals or beliefs. Moreover, religious groups can also offer community support and solidarity [60]. For both religious and non-religious individuals, nurse-counseling service and voluntary organizations support can also help to reduce distress associated with breast cancer diagnosis and treatment [56,61].

#### 5.2.3. Recreation

Patients are likely to be on long-term leave while recovering from treatment. Some patients may inevitably feel a sense of emptiness following a sudden break from their hectic work. As such, recreation becomes an important means to “kill time” and preoccupy them. Some forms of recreation include watching drama, going on excursions or trips, engaging in music therapy, pet therapy, or art therapy. Randomized controlled trials conducted on cancer patients have shown that music and art therapy are effective in reducing emotional distress and enhancing psychological well-being [62,63].

## 6. Holistic Needs after Treatment

Following treatment, breast cancer patients may suffer from physical side effects such as lymphedema, post-mastectomy pain syndrome, and post-chemotherapy cognitive impairment [64]. There may also be a persistent fear of cancer recurrence. It is hence imperative that doctors and patients work together to develop a personalized follow-up care plan for the coming months and years, so as to maintain good health, manage side effects, and monitor for cancer recurrence in both the native site and in other parts of the body. This could include scheduled doctor visits, mammograms, gynecological examinations, bone density exams, and others. In the management of breast cancer, the use of tools such as Mammaprint and 21-oncotype DX could also assist in predicting the risk of recurrence and making treatment decisions. Some of these tested genes are associated with proliferation (e.g., survivin and cyclin B1 and E2), invasion (e.g., matrix metalloproteinases 9 (MMP9) and cathepsin L2), estrogen (e.g., ER and PR), the human epidermal growth factor HER2/neu, and other hallmarks of cancers [65,66]. Furthermore, if a patient has a hereditary breast cancer, the management may vary in genetic testing. In hereditary breast cancer patients with abnormal BRCA1 or BRCA2 gene mutations, other female relatives may also be at risk of developing not just breast cancer, but also ovarian and other cancers. Other inheritable gene mutations such as TP53 in families with Li-Fraumeni syndrome, checkpoint kinase 2 (CHEK2), ataxia-telangiectasia mutated (ATM), E-cadherin (CDH1), serine/threonine kinase 11 (STK11), partner and localizer of BRCA2 (PALB2), and phosphatase and tensin homolog (PTEN), although rare, have been shown to increase the risk of early onset breast cancer [67,68]. These genes have been selected to be included in the panel of genes for genetic testing and some of these commercially available tests include BreastNext and Myriad myRisk [69]. In addition, while males are not as susceptible as females for breast cancer, those with a positive family history have a greater chance of contracting breast cancer, compared to other males in the general population [70]. Therefore, the family members of breast cancer patients with susceptibility genes, both male and female, may need to be counseled with respect to the risks of developing cancers associated with relevant genetic mutations. Besides genetic testing and counseling, psychosocial support may also be offered to such families. It should be noted that women with positive family histories of breast cancer tend to have a heightened sense of anxiety [71]. Appropriate genetic education and counseling would thus enable family members to fully appreciate the inherent uncertainty and implications behind genetic testing.

Patients may also worry about whether they can integrate back into the workplace and society after being on treatment for long periods of time. This is where support groups may prove to be beneficial. They may find solace in meeting people sharing similar situations as theirs and hearing from those who have recovered psychologically from breast cancer. For example, Singapore’s Breast Cancer Foundation runs the BCF Education and Empowerment Programme (BEEP) that engages not just survivors, but caregivers and medical personnel too [72]. Similarly, the Japanese breast cancer patients' association, Akebono-kai [73], helps women with breast cancer return to a normal life after treatment. In addition to providing social support, the Breast Cancer Care in the United Kingdom [74] also provides comprehensive information and education on coping with cancer.

Patients with advanced cancer may require long-term treatment while those with terminal cancer would require end-of-life care. The specific needs of these patient groups are not explored in this review. However, it must be noted that holistic needs care by the oncologist care team and the community is just as important, if not even more critical in determining quality of life for such patients.

## 7. Conclusions

Unlike in the past, breast cancer is not necessarily a death sentence. The past two decades have seen major advances in new surgical techniques, radiation approaches, and drugs for breast cancer, making it a physical illness that can potentially be fully cured. With such advances, the focus today should perhaps be on healing the patient holistically, taking into consideration their psychological, social, and spiritual well-being, and sometimes even treating the family too. Many hospitals are striving towards this goal by instituting a better holistic needs assessment and holistic care programs. Hence, for breast cancer patients, the diagnosis may be initially overwhelming, but the journey and nursing back to health need not be a lonely one.

## Figures and Tables

**Table 1:** The five stages of grief experienced by breast cancer patients.

Stages	Description
Denial	The first reaction to a malignant diagnosis is to deny the reality [37]. It is a temporary defense mechanism protecting one against the shock of a debilitating event [41].
Anger	As an individual is eventually forced to face the truth, she starts to question why this is happening to her, and who is to blame [37]. She experiences intense pain and helplessness that manifest as anger directed to those around them [41]. During this stage, people around them may develop emotional resentments which could worsen their relationship. It is thus important for family and friends to empathize with the individual’s grieving phases.
Bargaining	The individual focuses on what she or others could have done differently to prevent the cancer from occurring. She imagines how much better life could have been without cancer. She may also make a pact with God in the hope that life could go back to the times before the diagnosis [37]. While such thoughts may help the individual to accept the diagnosis, it could lead to an intense sense of guilt.
Depression	After accepting cancer as inevitable, the individual feels a sense of emptiness and profound sadness [37]. She may see no meaning in doing anything. While many people seek to avoid settling into this state, it is important to recognize depression as a natural response to a great loss. One has to let herself face her emotions upfront and feel her grief fully before she can recover completely. This stage should not be rushed and different individuals may spend varying amounts of time in this stage [41].
Acceptance	Acceptance may follow depression, as the individual decides that she is ready to accept what has happened [37]. She may not be completely alright with it, but she is willing to make adjustments and learn to live with it.
